# Laparoscopic Distal Gastrectomy for Duodenal Adenocarcinoma Located in a Duodenal Bulb Diverticulum: Report of a Rare Case

**DOI:** 10.70352/scrj.cr.25-0058

**Published:** 2025-06-28

**Authors:** Masato Nishimuta, Junichi Arai, Keiko Hamasaki, Yasumasa Hashimoto, Hayata Fukano, Tetsuro Tominaga, Takashi Nonaka, Keitaro Matsumoto

**Affiliations:** Department of Surgical Oncology, Nagasaki University Graduate School of Biomedical Sciences, Nagasaki, Nagasaki, Japan

**Keywords:** duodenal adenocarcinoma, duodenal bulb diverticulum, distal gastrectomy

## Abstract

**INTRODUCTION:**

Duodenal adenocarcinomas are relatively rare. We report here a particularly rare case of duodenal adenocarcinoma that arose within a duodenal bulb diverticulum and was successfully managed surgically.

**CASE PRESENTATION:**

A 70-year-old woman presented to us with a history of duodenal bulb ulceration and a diverticulum in the same area diagnosed by esophagogastroduodenoscopy. Routine endoscopic examination revealed an elevated lesion growing from within the diverticulum to outside of it. Examination of a biopsy specimen resulted in a diagnosis of well-differentiated adenocarcinoma. Computed tomography failed to detect any lesions in the duodenal bulb. No enlarged lymph nodes or distant metastases were found. Fluoroscopic examination revealed a pool of contrast suggestive of a diverticulum in the duodenal bulb on the anal side of the pyloric ring. Thus, the clinical stage according to the TNM Classification based on the 8th edition of the Union for International Cancer Control/American Joint Committee on Cancer (UICC/AJCC 8th edition) was T1N0M0: Stage I. Endoscopic resection was not feasible because the tumor’s origin was within the diverticulum. Furthermore, surgical local resection was considered not to be feasible because of the tumor size and location. We performed laparoscopic distal gastrectomy, D1+ lymph node dissection, and Roux-en-Y reconstruction. The patient was discharged on the 10th postoperative day and is currently on outpatient follow-up with no evidence of recurrence 1 year postoperatively.

**CONCLUSIONS:**

We report here a rare case of an adenocarcinoma arising within a duodenal bulb diverticulum. We successfully performed a laparoscopic distal gastrectomy, this procedure being considered the optimal surgical approach for this patient.

## INTRODUCTION

Duodenal cancers have been considered rare, reportedly accounting for only 0.4% of gastrointestinal cancers.^1)^ However, reports from North America and Europe indicate that the prevalence of duodenal cancer is increasing.^2,3)^ In Japan, the rate is 23.7 per million population, which is higher than in Europe and the USA.^4)^ Endoscopic treatment is recommended for duodenal carcinomas that have not infiltrated beyond the mucosa.^5)^ This procedure requires advanced endoscopic techniques. Surgical resection is sometimes required, depending on the site and size of the tumor. We report here a case of duodenal carcinoma arising within a duodenal bulb diverticulum, an extremely rare phenomenon, which we completely resected by laparoscopic distal gastrectomy.

## CASE PRESENTATION

**Fig. 1:**
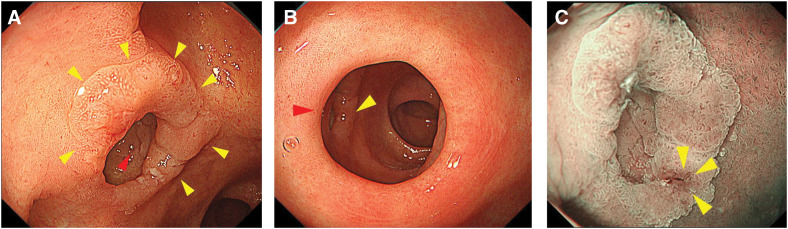
Findings of esophagogastroduodenoscopy. (**A**) An elevated lesion (yellow arrowheads) spread from within the diverticulum (red arrowhead) to the surrounding area. (**B**) The image shows the relationship between the pyloric ring (red arrowhead) and the tumor (yellow arrowhead). It can show that the tumor is located just on the anal side of the pyloric ring. (**C**) Narrow-band imaging (NBI) mode. The disturbed vascular structure (yellow arrowheads) suggests the presence of a malignant tumor.

**Fig. 2:**
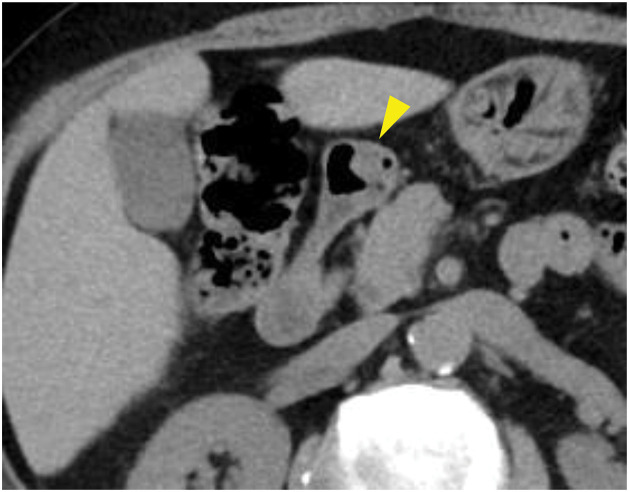
Findings of computed tomography. Computed tomography scan image. No lesions were detected in the duodenal bulb (yellow arrowhead).

**Fig. 3:**
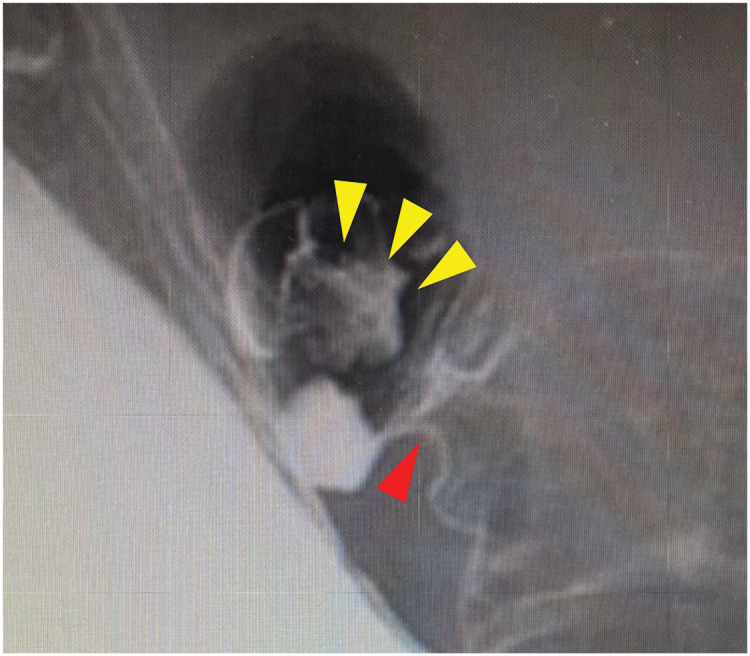
Findings of gastric fluoroscopic examination. A pool of contrast (yellow arrowheads) suggestive of a diverticulum in the duodenal bulb on the anal side of the pyloric ring (red arrowhead).

A 73-year-old woman had a history of an ulcer on the anterior wall of the duodenal bulb and was subsequently found by esophagogastroduodenoscopy to have a diverticulum in the same location. During a follow-up esophagogastroduodenoscopy, an elevated lesion that had spread from within the diverticulum to the surrounding area was detected (**Fig. 1**). A duodenal adenocarcinoma was diagnosed by pathological examination of an endoscopic biopsy. A computed tomography scan failed to reveal any lesions in the duodenal bulb (**Fig. 2**). No enlarged lymph nodes or distant metastases were found. Fluoroscopic examination revealed a pool of contrast suggestive of a diverticulum in the duodenal bulb on the anal side of the pyloric ring (**Fig. 3**). Based on these findings, the clinical stage according to the TNM Classification based on the 8th edition of the Union for International Cancer Control/American Joint Committee on Cancer (UICC/AJCC 8th edition) was T1N0M0: Stage I.

We discussed treatment options with a gastrointestinal endoscopist. First, endoscopic resection was considered unfeasible because the tumor had originated within a diverticulum. Second, it was considered that local resection of the duodenum including laparoscopy and endoscopy cooperative surgery (LECS) was inadvisable because the location and size of the tumor posed an unacceptable risk of postoperative stricture and deformity. Third, because the tumor was within a diverticulum, it was considered that the preoperative estimate of depth may have been inaccurate and the tumor may have infiltrated beyond the submucosa, mandating lymph node dissection around the pylorus. Finally, a decision was made to perform distal gastrectomy and lymph node dissection.

**Fig. 4:**
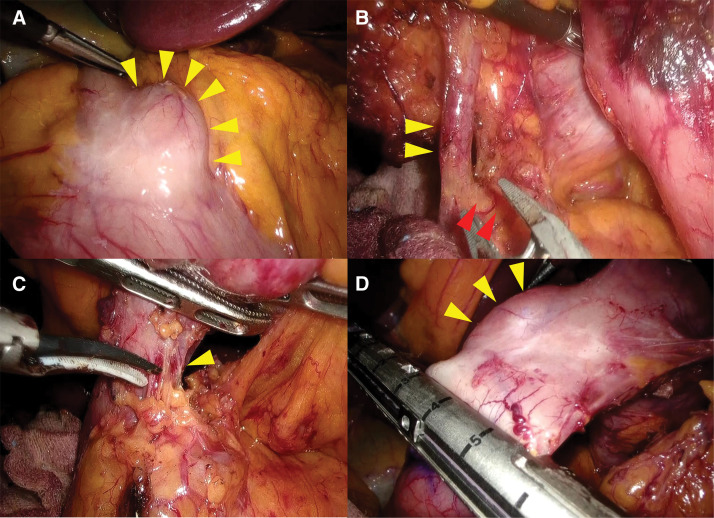
Intraoperative findings. (**A**) Findings at the start of surgery. A diverticulum (yellow arrowheads) was observed in the anterior wall of the duodenal bulb. (**B**) Findings of No. 6 lymph node dissection. The right gastroepiploic artery (yellow arrowheads) were dissected at the root. The red arrowheads are the preserved gastroduodenal artery. (**C**) To expose the duodenal dissection line, a branch of the superior pancreaticoduodenal artery (yellow arrowheads) was dissected and separated from the pancreatic head. (**D**) The duodenum was dissected on the anorectal side of the diverticulum (yellow arrowheads) while observing with an intraoperative endoscope.

We therefore performed laparoscopic distal gastrectomy (**Fig. 4**), D1+ lymph node dissection, and Roux-en-Y reconstruction. A diverticulum protruding from the intraperitoneal side into the anterior wall of the duodenal bulb was observed. Lymph nodes 5 and 6 dissection was performed in accordance with the Gastric Cancer Treatment Protocol. To expose the duodenal dissection line, a branch of the superior pancreaticoduodenal artery was dissected and separated from the pancreatic head. The duodenum dissection line was determined by intraoperative endoscopy while looking at the tumor anal side. The operative time was 341 minutes and blood loss was 20 mL. The postoperative course was uneventful and the patient was discharged on postoperative day 10.

**Fig. 5:**
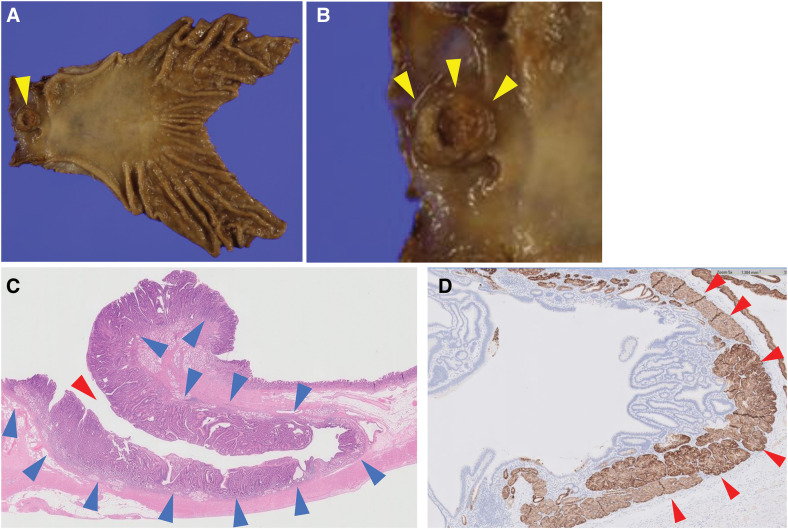
Operative specimen. (**A**) Macroscopic findings. A tumor (yellow arrowhead) is located in the duodenal bulb. R0 resection was achieved. (**B**) Enlarged macroscopic findings (yellow arrowheads). (**C**) Photomicrograph of hematoxylin and eosin-stained specimen showing atypical cells in the area indicated by the blue arrowheads, extending from within the diverticulum (red arrowhead) to outside of it. (**D**) Photomicrograph of specimen immunostained with desmin (red arrowheads) showing that there is an intrinsic muscular layer.

The pathological findings are shown in **Fig. 5**. The tumor size was 15 × 16 mm. The adenocarcinoma extended from within the diverticulum to the surrounding area but remained intramucosal (**Fig. 5C**). Immunostaining with desmin showed that the diverticulum in the duodenal bulb had an intrinsic muscular layer (**Fig. 5C**), indicating that it was a true diverticulum. No lymph node metastases were detected. The pathological stage according to the TNM classification (UICC/AJCC 8th edition) was T1N0M0: Stage I. 1 year postoperatively, the patient has no evidence of recurrence.

## DISCUSSION

To the best of our knowledge, this is the first reported case of an adenocarcinoma arising within a diverticulum in the duodenal bulb. Duodenal diverticulae are relatively common, with the reported prevalence being 5%–32%.^6,7)^ However, they most commonly occur in the second part of the duodenum and rarely in the duodenal bulb.^8)^ Our patient had a history of taking medications for ulceration of the duodenal bulb. We postulated that the duodenal ulceration had resulted in the formation of the diverticulum.

A search of PubMed revealed no reports of tumors arising within a diverticulum in the duodenal bulb. Our team made a treatment plan in conjunction with a gastrointestinal endoscopist. The tumor appeared to be confined to the mucosa within the observable range; thus, endoscopic treatment was considered.^5,9)^

There are several reports on safe techniques for endoscopic resection of tumors located close to duodenal diverticula.^10,11)^ However, endoscopic treatment was deemed to be contraindicated in our patient’s case because most of her tumor was located within the diverticulum. Local duodenal resection without lymph node dissection is a treatment option for duodenal intramucosal carcinomas that are difficult to resect endoscopically.^5)^ However, because of the size of the postoperative defect, the risk of deformation and stenosis of the duodenal bulb after local resection was considered unacceptably high in our patient’s case.

We also concluded that the clinical estimate of depth of penetration was likely inaccurate because the center of the tumor was hidden within the diverticulum; it has been reported that the tumor depth should be assumed to be deeper than estimated clinically for colon cancers arising from within a diverticulum.^12,13)^ We therefore considered that our patient’s tumor likely extended into the submucosa. According to the Japanese guidelines^5)^ for duodenal cancer, the frequency of lymph node metastasis is 5%–11% for duodenal cancers with submucosal invasion. The standard procedure for duodenal cancer with submucosal invasion is pancreatoduodenectomy. In the duodenal bulb, the peripyloric lymph nodes (Nos 5 and 6) are considered as sentinel nodes.^14,15)^ Thus, distal gastrectomy is considered to be one of the surgical treatment options for tumors confined to the duodenal bulb.

For the above reasons, we considered that distal gastrectomy was the optimal procedure for our patient. The pathological diagnosis was intramucosal carcinoma.

## CONCLUSIONS

We have presented here a case of intramucosal adenocarcinoma arising within a diverticulum in the duodenal bulb. We concluded that distal gastrectomy was the optimal procedure in this case.

## ACKNOWLEDGMENTS

We thank Dr Trish Reynolds, MBBS, FRACP, from Edanz (https://jp.edanz.com/ac) for editing a draft of this manuscript.

## DECLARATIONS

### Funding

This research did not receive any specific grants from funding agencies in the public, commercial, or not-for-profit sectors.

### Authors’ contributions

MN and JA conceptualized the study.

KH, YH, HF, and TT collaborated in patient care.

TN and KM provided input on the manuscript.

All authors have read and approved the final manuscript.

### Availability of data and materials

The data that support the findings of this study are available from the corresponding author upon reasonable request.

### Ethics approval and consent to participate

This work does not require ethical considerations or approval. Informed consent to participate in this study was obtained from the patient.

### Consent for publication

Written, informed consent was obtained from the patient for publication of this case report.

### Competing interests

The authors declare that they do not have any competing interests.
